# Efficacy and safety of PD-1 inhibitors in recurrent or metastatic squamous cell carcinoma of the head and neck: a systematic review and meta-analysis

**DOI:** 10.3389/or.2026.1745071

**Published:** 2026-06-05

**Authors:** Abdullah Alabbasi, Muhanad Alzahrani, Ziyad Alzahrani, Faris Sultan, Maha AlQahtani, Alaa Sultan

**Affiliations:** 1 First Cluster, Joint Program of Family Medicine Jeddah, Jeddah, Saudi Arabia; 2 College of Medicine, King Saud bin Abdulaziz University for Health Sciences, Jeddah, Saudi Arabia; 3 King Abdullah International Medical Research Center, Jeddah, Saudi Arabia; 4 Department of Surgery, College of Medicine, King Saud University, King Saud University Medical City, Riyadh, Saudi Arabia; 5 College of Medicine, King Saud University, Riyadh, Saudi Arabia; 6 Plastic and Reconstruction Surgery Department, Umm Al-Qura University College of Medicine, Mecca, Saudi Arabia

**Keywords:** HNSCC, meta-analysis, metastasis, PD-1, review, systematic

## Abstract

**Background:**

Conventional cytotoxic regimens for recurrent or metastatic head and neck squamous cell carcinoma (R/M HNSCC) offer limited durable benefit and are associated with significant toxicity. Immune checkpoint inhibitors targeting the PD-1/PD-L1 pathway have emerged as alternative treatments; however, variability in response, survival, and toxicity across studies necessitates comprehensive synthesis. This systematic review and meta-analysis evaluated the efficacy and safety of PD-1 inhibitors in adults with R/M HNSCC.

**Methods:**

A systematic search of PubMed (MEDLINE), Embase, Scopus, and Cochrane databases was conducted. Study selection followed the PICOS framework and PRISMA guidelines, with screening performed using Rayyan. Data synthesis was conducted in R (version 4.1.1) using the meta and metafor packages. The protocol was registered in PROSPERO (CRD420251169189).

**Results:**

Fourteen studies (n = 1,231) were included: nine in non-NPC HNSCC (primary analysis) and five NPC-only studies (sensitivity subgroup). In non-NPC HNSCC, pooled ORR was 22% (95% CI: 8%–48%, I^2^ = 87%) for combination therapy and 14% (95% CI: 3%–46%, I^2^ = 29%) for monotherapy. For combination regimens, pooled best responses were: CR 5.6%, PR 21.7%, SD 28.2%, and PD 34.9%. One-year PFS was 29.3% (95% CI: 13.5%–52.4%, I^2^ = 88%, k = 3), and 1-year OS was 59.0% (95% CI: 42.3%–73.8%, I^2^ = 82%, k = 8). Grade 3–5 treatment-related adverse events occurred in 16.3% of monotherapy patients and 48.2% of combination therapy patients. In NPC-only studies (k = 5), pooled ORR was 61.1% (95% CI: 13.4%–94.1%, I^2^ = 89%). Estimates are descriptive and largely derived from single-arm studies with substantial heterogeneity.

**Conclusion:**

PD-1 inhibitor combinations may be associated with higher response rates and 1-year survival compared with monotherapy in non-NPC HNSCC, at the cost of increased toxicity. These findings are hypothesis-generating and do not establish causal efficacy. Well-designed randomized trials stratified by disease subtype and treatment context are needed, alongside greater focus on quality-of-life outcomes.

## 1 Introduction

The term head and neck squamous cell carcinoma (HNSCC) describes a class of cancers that develop from the squamous cells that line the tissues of the head and neck area, such as the salivary glands, nasal cavity, lip, paranasal sinuses, nasopharynx, oropharynx, oral cavity, and hypopharynx (1). The incidence and mortality rates of HNSCC vary significantly depending on geographic location and demographic variables, making it a major global health concern (1). According to the most recent GLOBOCAN estimates (2022), HNSCC is the seventh most common cancer worldwide, accounting for an estimated 480,000 annual fatalities and 940,000 new cases globally (2). The incidence rates in the USA have risen by 15.5%, according to recent epidemiological data (3). As a result of this development, HPV-related diseases have supplanted tobacco-related HNSCC, with oropharyngeal illness expected to overtake oral disease (4). Chronic oral trauma, various types of chewing, alcohol use, and smoking are other known risk factors (5).

Head and neck cancer patients experience a variety of symptoms, including psychological symptoms such as depression, irritability, and loss of self-esteem, as well as physical symptoms related to diet and feeding, communication problems, pain, and overall health (6). Patients who have been treated for HNSCC are at risk of developing the disease again at distant locations or at previous disease sites (7). In this context, thorough documentation of recurrence and metastatic spread patterns is a prerequisite for the development of evidence-based posttreatment surveillance guidelines for recurrent/metastatic head and neck squamous cell carcinoma (R/M HNSCC) (7). However, only a small number of therapeutic modalities have been demonstrated to improve overall survival (OS) or progression-free survival (PFS), and patients with metastases tend to remain incurable (8).

Growing evidence highlights that the percentage of locoregional failure is approximately 40%–50%, and the percentage of distant failure is 20%–30% in patients with locoregionally advanced HNSCC who have received upfront surgery or upfront chemoradiation (9, 10). Consequently, approximately 50% of patients with HNSCC develop recurrent or metastatic disease. Recurrent/metastatic HNSCC has a poor prognosis and a median survival of approximately 12 months, making it a challenging disease to treat (11). Over the years, a number of therapeutic approaches have been developed, including monochemotherapy, polychemotherapy, the combination of chemotherapy and cetuximab, and, more recently, *“new generation immunotherapy,”* which makes use of immune checkpoint inhibitors (ICIs) (12). Programmed cell death protein-1 (PD-1) receptors, programmed death-ligand 1 (PD-L1) receptors, and cytotoxic T lymphocyte-associated protein-4 (CTLA-4) are the major established targets for cancer immunotherapy with ICIs (13). Given that the predominant mechanism involves dysregulated immune signaling pathways (14), which may suppress antitumor immune responses in HNSCC (15), inhibition of the PD-1/PD-L1 axis has emerged as a critical therapeutic strategy. The interaction between PD-1 on T cells and its ligand PD-L1 on tumor or immune cells leads to T-cell inactivation and facilitates tumor immune evasion.

Conventional cytotoxic regimens offer limited long-term benefit and are often associated with substantial toxicity. In recent years, immune checkpoint inhibitors that target the programmed cell death-1 (PD-1)/programmed death-ligand 1 (PD-L1) pathway, such as nivolumab, pembrolizumab, and cemiplimab, have transformed the treatment landscape of advanced solid tumors, including R/M HNSCC. By restoring antitumor T-cell activity and reversing immune exhaustion, PD-1 inhibitors can induce durable responses in a subset of patients who otherwise have limited therapeutic options. However, variability in response rates, survival benefits, and toxicity profiles across clinical trials highlights the need for a comprehensive synthesis of available evidence. The current systematic review and meta-analysis aimed to quantitatively evaluate the efficacy and safety of PD-1 inhibitors in adults with R/M HNSCC, identify the line of therapy and treatment setting, and compare the outcome variables between monotherapy and combination therapy regimens. Prior systematic reviews and meta-analyses in this field have addressed broad efficacy of immune checkpoint inhibitors in R/M HNSCC; however, several knowledge gaps remain unaddressed. First, most prior syntheses do not explicitly separate NPC from non-NPC HNSCC, despite their biological and clinical distinctions. Second, the distinction between monotherapy and combination therapy outcomes is inconsistently handled in terms of statistical frameworks (pooled proportions vs. comparative effect sizes). Third, regimen-class-specific safety stratification and the role of exploratory biomarkers have not been systematically examined with appropriate interpretive guardrails. The present review addresses these gaps by separately analyzing monotherapy and combination therapy, distinguishing NPC and non-NPC populations, and framing biomarker findings explicitly as hypothesis-generating.

## 2 Methodology

### 2.1 Eligibility criteria

The PRISMA guidelines and the ‘Population, Intervention, Comparison, Outcome, and Study Design (PICOS)’ scheme were utilized to generate the eligibility criteria (16). This systematic review was prospectively registered in PROSPERO (CRD420251169189). Studies were considered eligible for inclusion if they were published between January 2000 and December 2025. The target population included adults (≥18 years) with histologically confirmed recurrent or metastatic squamous cell carcinoma of the head and neck (oral cavity, oropharynx, hypopharynx, and larynx). Mixed cohorts were eligible if R/M HNSCC data were reported separately. Nasopharyngeal carcinoma (NPC), although historically classified under HNSCC, is biologically and clinically distinct. Studies involving NPC were retained in the overall dataset but are presented as a distinct subgroup throughout this review; all primary conclusions are based on non-NPC HNSCC studies, with NPC studies reported separately in sensitivity analyses. The PICOS scheme for the current study is further elaborated in the table below. The inclusion criteria were developed carefully after the development of the PICOS criteria for the systematic review (Table 1).

**TABLE 1:** PICOS framework for the literature search.

Category	Inclusion criteria	Exclusion criteria
Population	Adults (≥18 years) with histologically confirmed recurrent or metastatic squamous cell carcinoma of the head and neck (oral cavity, oropharynx, hypopharynx, larynx). Mixed cohorts were eligible if R/M HNSCC data were reported separately	Pediatric populations, non-HNSCC histologies (e.g., nasopharyngeal or salivary gland cancers), or studies without separable R/M HNSCC outcomes
Intervention	PD-1 inhibitors (nivolumab, pembrolizumab, cemiplimab) given as monotherapy or in combination with other systemic agents	PD-L1 inhibitors without PD-1 agents, or studies evaluating nonimmunotherapy interventions
Comparator	Placebo, best supportive care, standard chemotherapy, other immunotherapy, or historical control arms	Studies without a comparator or without a PD-1–based comparison when outcomes were nonextractable
Outcomes	Overall survival (OS) and objective response rate (ORR) as primary outcomes; secondary outcomes included progression-free survival (PFS), disease control rate (DCR), duration of response (DOR), quality of life (QoL), and treatment-related or immune-related adverse events (grade ≥3)	Studies not reporting at least one prespecified outcome or lacking extractable clinical data
Study design	Randomized controlled trials (RCTs), including both single- and double-arm interventional designs (phase II or III)	Observational, retrospective, preclinical, case series (<10 patients), reviews, or editorials
Timeframe	Studies published between 2000 and 2025	Publications outside this range
Language	English-language full-texts	Non-English studies without available English translation

### 2.2 Information sources

We searched the following bibliographic databases from 1 January 2000 through 31 December 2025: MEDLINE (via PubMed), Embase, the Cochrane Central Register of Controlled Trials (CENTRAL), Web of Science, and Scopus. The clinical trial registries searched included ClinicalTrials.gov. In addition, targeted searches were performed in high-impact journals and publisher platforms (for example, JAMA Network Open and Elsevier journals). When outcome data were incomplete or unclear, we contacted the study authors or trial sponsors to request missing information. All searches were restricted to English-language records.

#### 2.2.1 Search strategy

The search strategy was devised following the PICOS scheme to retrieve pertinent data from digital databases. In the final sample, 14 studies (from a total sample of n = 214) met the eligibility criteria. A search query was formulated encompassing the following keywords:

“((“Squamous Cell Carcinoma of Head and Neck”[Mesh] OR “Head and Neck Neoplasms”[Mesh] OR.

(Head and neck squamous cell carcinoma[tiab]) OR (HNSCC[tiab]) OR (“recurrent”[tiab] OR “metastatic”[tiab]) AND (“head and neck cancer”[tiab] OR “squamous cell carcinoma”[tiab])) AND (“Programmed Cell Death 1 Receptor”[Mesh] OR “PD-1 inhibitor”[tiab] OR “PD1 inhibitor”[tiab] OR (“nivolumab”[tiab] OR “pembrolizumab”[tiab] OR “cemiplimab”[tiab]) OR (“Opdivo”[tiab] OR “Keytruda”[tiab] OR “Libtayo”[tiab])) AND (randomized controlled trial[pt] OR randomized[tiab] OR randomized[tiab] OR “phase II”[tiab] OR “phase III”[tiab]) AND (“2000/01/01”[Date - Publication]: “2025/12/31”[Date - Publication]) AND (english[lang])” Selection process.

The study methodology was developed on the basis of a review of relevant peer-reviewed literature. Articles meeting the predefined inclusion criteria were appraised via the PICOS framework to ensure methodological strength. The screening and selection process was facilitated via **Rayyan.ai** was used solely to manage records and facilitate blinded screening; no AI-generated inclusion/exclusion recommendations were used for eligibility decisions. Two reviewers independently screened titles/abstracts and full texts using prespecified criteria. Disagreements were resolved by consensus or, if unresolved, by arbitration with a third reviewer (17). A total of 14 studies were found to be appropriate for inclusion. Articles were excluded if they targeted a non-relevant population (e.g., pediatric patients or non-HNSCC histologies), used an inappropriate study design (observational, retrospective, case series <10 patients, preclinical, reviews, or editorials), lacked extractable efficacy or safety outcomes (OS, PFS, ORR, TRAEs), or reported mixed cancer cohorts without separable R/M HNSCC data. Studies outside the prespecified timeframe or without English-language full texts were also excluded. In several cases, studies were excluded for more than one of these reasons.

### 2.3 Data items

Two reviewers independently extracted the data via a standardized form. The extracted variables included study characteristics, patient demographics, interventions, comparators, and outcomes. Where the data were incomplete, the study authors or trial sponsors were contacted for clarification. The extracted information included publication details (first author, year of publication, journal, country, funding source, and clinical trial registration number) and study characteristics (design type [randomized controlled, nonrandomized, or single-arm], sample size, and reported inclusion and exclusion criteria). Population characteristics, including median or mean age, sex distribution, Eastern Cooperative Oncology Group (ECOG) performance status, primary tumor site, HPV/p16 status, PD-L1 expression assay and cutoff, prior systemic therapies, and treatment line, were recorded. Intervention details included the specific PD-1 inhibitor used, dosing regimen, treatment schedule, duration of therapy, and combination agent. For the comparator arms, data were extracted on drug/regimen type, dosing, and schedule. The outcomes of interest included overall survival (OS; hazard ratio, 95% confidence interval, and p value), progression-free survival (PFS), the objective response rate (ORR; numerator and denominator), the disease control rate (DCR), the duration of response (DOR), quality of life (QoL) measures and instruments used, and safety outcomes. Safety data included all-grade and grade ≥3 treatment-related adverse events, immune-related adverse events, and treatment discontinuation due to toxicity. The median follow-up time and censored information were also collected.

**FIGURE 1:**
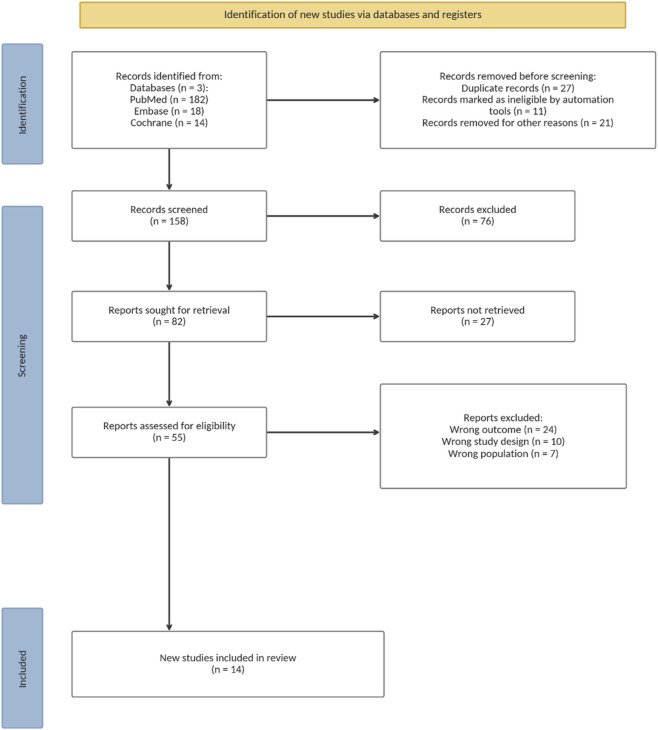
PRISMA chart for the systematic review.

After the secondary screening process was complete, we assessed the overall sample size (n = 14) of the selected studies. To create a PRISMA flow chart that follows the rules of the Preferred Reporting Items for Systematic Review and Meta-Analysis (PRISMA), we used articles from reputable journals and other sources (18). The study selection process is summarized in the PRISMA flow diagram (Figure 1).

### 2.4 Risk of bias

**FIGURE 2:**
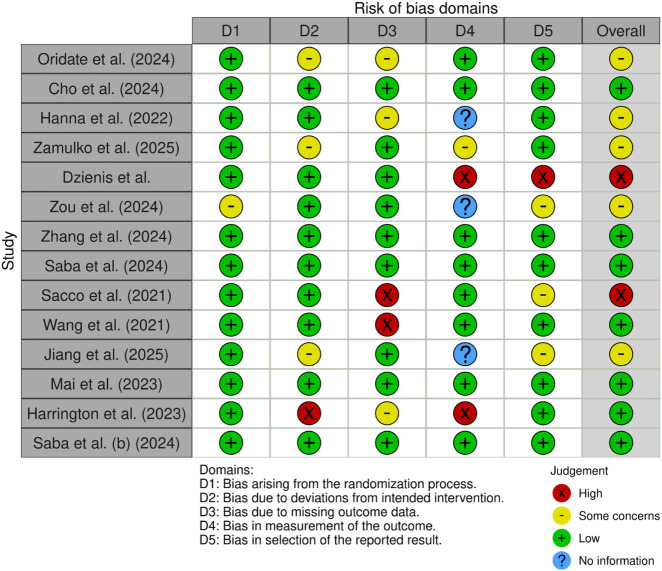
Traffic light plot for risk of bias across the included studies.

**FIGURE 3:**
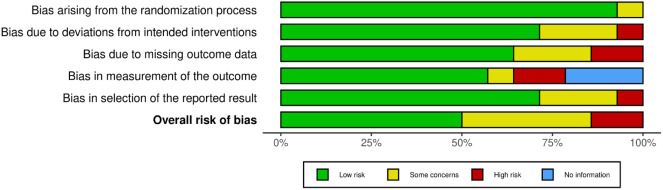
Summary plot for risk across the individual domains of bias.

We conducted a comprehensive analysis of bias in each study selected for quality assessment. Randomized controlled trials were assessed via the Cochrane RoB 2 tool across five domains (randomization process, deviations from intended interventions, missing outcome data, measurement of the outcome, and selection of the reported result). Non-randomized and single-arm interventional studies were appraised narratively and, where applicable, with ROBINS-I. Figures 2, 3 present traffic-light and summary plots for randomized trials only; non-randomized studies are described separately in the text (19).

### 2.5 Standardization of non-poolable data

To ensure consistency in data synthesis and enable quantitative pooling in meta-analysis, when studies reported continuous outcomes as the means and corresponding confidence intervals (CIs), standard deviations (SDs) were derived via established statistical methods. Specifically, the standard error (SE) was calculated from the CI bounds via the following formula:

$$SE=\left(Upper\;CI-Lower\;CI\right)\;/\;\left(2\times t\alpha /2,df\right)$$

where tα/2,df ​ represents the critical value from the Student’s t-distribution for the given confidence level and degrees of freedom (df = n−1). The SD was then obtained by multiplying the SE by the square root of the sample size SD=SE×√n . This approach is consistent with guidance from the Cochrane Handbook for Systematic Reviews of Interventions (version 6.4, Section 6.5.2.3) for converting summary statistics when raw data are unavailable (20).

### 2.6 Statistical analysis

All the data in the investigation were available as dichotomous variables. The pretest and posttest interventions were extracted for continuous outcomes. Owing to a lack of paired t-test data, “n/N” (frequency of events/total number of events) was used independently in crossover studies. In the meta-analysis, several statistics were used to assess the heterogeneity of the included papers. The effect size varied among studies, as indicated by the tau square (τ^2), which is an indication of the within-study variance. Degrees of freedom (dfs) represent the number of independent comparisons required to calculate the pooled effect size. If detected differences in effect sizes between studies were greater than what would be expected by random chance, they were determined via the chi-square (χ2) test. If the chi-square value was significant, heterogeneity was considered positive. The fraction of total variation that can be attributed to heterogeneity rather than random variation was assessed by the I-square (I^2^). Since statistical heterogeneity was assessed via the I^2^ statistic, values above 50% were indicative of substantial heterogeneity. Elevated values suggested increased variability and disparity across research findings. The “forest plots” were used to represent the analysis, with a null-effect line on the central axis, and the “diamond” represents the combined effect of individual studies. All pooled estimates derived from single-arm studies represent descriptive observed proportions and should not be interpreted as causal treatment effects; comparative inference is reserved for outcomes with a concurrent randomized control arm. All pooled estimates derived from single-arm studies represent descriptive observed proportions and should not be interpreted as causal treatment effects; comparative inference applies only where a concurrent randomised control arm exists. RevMan (version 5.4.1) facilitated the analysis of the 14 primary studies (21).

### 2.7 Safety outcomes and harmonization

Safety outcomes were defined as treatment-related adverse events (TRAEs) of any grade and grade ≥3, following the Common Terminology Criteria for Adverse Events (CTCAE) as reported by individual studies. Where studies used grade 3–5 *versus* grade ≥3 definitions interchangeably, these were harmonized to a grade ≥3 classification for pooling purposes. Studies reporting TRAEs in a manner incompatible with this definition were included in a structured narrative summary rather than quantitative pooling. Safety analyses were stratified by regimen class (monotherapy vs. combination therapy) to avoid masking regimen-specific signals; pooled safety estimates across the combined dataset should be interpreted with caution given the heterogeneity in regimen composition and reporting standards across included studies.

## 3 Results

### 3.1 Characteristics of the included studies

A total of 14 studies were synthesized for this review, encompassing a range of trial designs and geographical settings. The methodologies primarily consisted of phase 3 randomized controlled trials (n = 4), phase 2 single-arm trials (n = 8), one phase 2 nonrandomized trial, and one single-arm phase IV study. The clinical setting for most included studies was recurrent or metastatic squamous cell carcinoma of the head and neck (R/M HNSCC) or recurrent or metastatic nasopharyngeal carcinoma (RM-NPC). Two investigations focused on locoregionally recurrent disease suitable for salvage surgery or reirradiation. Of the 14 included studies, five (studies 6, 7, 10, 11, and 12: Zou et al., Zhang et al., Wang et al., Jiang et al., and Mai et al.) enrolled exclusively NPC populations. Given that NPC is biologically distinct from non-NPC HNSCC—with unique EBV-driven pathogenesis, endemic geographic distribution, and distinct chemosensitivity—these studies are reported as a separate subgroup throughout the meta-analysis. The primary pooled estimates presented in this review reflect non-NPC HNSCC populations; NPC study data are presented separately and should be interpreted in that distinct clinical context. Geographical implementation was distributed across multinational centers, notably, global phase 3 trials, specific Japanese subgroup analyses, and several RM-NPC trials conducted primarily in China and Asia. Patient sample sizes varied significantly, ranging from a minimum of 10 patients in the prematurely terminated niraparib/dostarlimab study to a maximum of 425 patients in the total enrolled population of the CheckMate 714 trial, with an aggregated total of 1,231 participants across all studies. The most frequently measured outcomes reported across the 14 studies were the objective response rate (ORR), progression-free survival (PFS), and overall survival (OS). A summary of the outcomes measured across the included studies is provided in the table below (Table 2).

**TABLE 2:** Outcomes assessed across the included studies.

Sr	Author (Year)	Type of cancer	Stage of cancer	Extent of cancer	Agent/Regimen used	Primary outcomes	Secondary outcomes
1	(22)	Head and neck squamous cell carcinoma (HNSCC)	R/M (Recurrent/Metastatic)	Incurable by local therapy	1. Pembrolizumab; 2. Pembrolizumab + chemotherapy; 3. EXTREME (cetuximab + chemotherapy)	Overall survival (OS) and progression-free survival (PFS)	Objective response rate (ORR), duration of response (DOR), and safety
2	(23)	Head and neck squamous cell carcinoma (HNSCC)	R/M	Measurable disease (RECIST v1.1). 81% metastatic disease overall	1. Pembrolizumab + epacadostat; 2. Pembrolizumab monotherapy; 3. EXTREME regimen	Objective response rate (ORR) (investigator assessment)	Safety and tolerability; change in serum kynurenine (exploratory)
3	(24)	Squamous cell carcinoma of the head and neck (HNSCC)	Locoregionally recurrent. Majority were clinical stage III or IV at trial entry (89%)	Recurrent, resectable locoregionally	Nivolumab (N) + lirilumab (L, anti-KIR)	1-year disease-free survival (DFS) from salvage surgery	Safety, tolerability, preop radiologic response (ORR), overall survival (OS)
4	(25)	Head and neck squamous cell carcinoma (HNSCC)	R/M	60% distant metastasis (M1)	Niraparib (PARPi) + dostarlimab (TSR-042, anti-PD-1)	Overall response rate (ORR) and clinical benefit (CR, PR, or SD) by RECIST v1.1	N/A (single-arm trial designed to test a 50% clinical benefit threshold based on historical PD-1 monotherapy rates)
5	(26)	Head and neck squamous cell carcinoma (HNSCC)	R/M. 92% stage IV.	68% M1 (distant metastasis)	Pembrolizumab + carboplatin + paclitaxel	Objective response rate (ORR) per RECIST v1.1 by BICR	Standard-of-care regimen (pembro + platinum + fluorouracil [FU])
6	(27)	Nasopharyngeal carcinoma (NPC)	Recurrent or metastatic (R/M)	52.4% locoregional recurrence only; 47.6% distant metastasis	Gemcitabine + toripalimab (anti-PD-1)	Incidence of grade 3 adverse events (AEs)	N/A (single-arm)
7	(28)	Nasopharyngeal carcinoma (NPC)	Recurrent or metastatic (R/M)	Failed at least one line of platinum-based therapy	Toripalimab (anti-PD-1) + anlotinib (TKI)	N/A (ORR/PFS/OS evaluated)	Safety, PFS, OS, ctDNA biomarker analysis
8	(29)	Head and neck squamous cell carcinoma (HNSCC)	R/M	Inoperable	Pembrolizumab (anti-PD-1) + cabozantinib (VEGFR-TKI)	Overall response rate (ORR), safety, and tolerability	Progression-free survival (PFS), overall survival (OS), correlative biomarkers (p-MET, TIME)
9	(30)	Head and neck squamous cell carcinoma (HNSCC)	Recurrent or metastatic (R/M)	Not amenable to curative intent local therapy	Pembrolizumab (PD-1 inhibitor) + cetuximab (EGFR inhibitor)	Overall response rate (ORR) by 6 months	12 months PFS rate, PFS, OS, DOR, safety, tolerability
10	(31)	Nasopharyngeal carcinoma (NPC)	R/M. 93.1% stage IVb	Refractory to standard therapy	Toripalimab (anti-PD-1)	Objective response rate (ORR) determined by IRC per RECIST v1.1	Safety, duration of response (DOR), PFS, OS, DCR, EBV DNA, TMB
11	(32)	Recurrent or metastatic nasopharyngeal carcinoma (RM-NPC)	Initial stage (AJCC 8th edition): II–III (36%), IVa (40%), IVb (24%)	Refractory to at least one-line prior chemotherapy and anti-PD-1 systemic therapy. 52% had distant lymph node metastases.	Cadonilimab (PD-1/CTLA-4 bispecific antibody) + TPC chemotherapy (NAB-paclitaxel, cisplatin/lobaplatin, and capecitabine)	Objective response rate (ORR) (proportion of patients with CR or PR per RECIST V1.1)	Progression-free survival (PFS), overall survival (OS), duration of response (DoR), median time to response (mTTR), and safety
12	(33)	Recurrent or metastatic nasopharyngeal carcinoma (RM-NPC)	R/M	42% primary metastatic; 58% recurrent	Toripalimab (anti-PD-1 monoclonal antibody) vs. placebo, both combined with gemcitabine-cisplatin chemotherapy	Progression-free survival (PFS) as assessed by a blinded independent central review (BICR)	Overall survival (OS), objective response rate (ORR), duration of response (DOR), safety
13	(34)	Recurrent or metastatic squamous cell carcinoma of the head and neck (R/M HNSCC)	R/M	Platinum-refractory R/M HNSCC	1. Nivolumab + ipilimumab (dual ICB); 2. Nivolumab (anti-PD-1) + placebo	Objective response rate (ORR) and duration of response (DOR) by BICR	PFS, OS, safety
14	(35)	Recurrent or second primary head and neck squamous cell carcinoma (HNSCC)	Locoregionally Recurrent/Second primary	Nonmetastatic, arising within a previously irradiated field	Nivolumab (anti-PD-1) + Intensity-modulated reirradiation therapy (IMRT)	Progression-free survival (PFS)	OS, incidence/types of toxic effects, patient-reported outcomes

### 3.2 Primary outcomes

Across the 14 studies, 9 demonstrated a positive effect or improved response to treatment with PD-1 agents or combinations compared with controls or historical data, two showed a negative effect or met futility criteria for the primary endpoint, and three exhibited a mixed or no significant primary benefit.

### 3.3 The study-specific results demonstrated highly varied efficacy

The JUPITER-02 phase III trial demonstrated a robust positive effect, finding that toripalimab plus chemotherapy significantly prolonged median PFS (21.4 months vs. 8.2 months; HR, 0.52) and OS (HR, 0.63) compared with chemotherapy alone in R/M NPC patients, with a higher ORR (78.8% vs. 67.1%) (33). In the KEYNOTE-048 Japanese subgroup analysis, pembrolizumab monotherapy improved OS over EXTREME chemotherapy in the total population (22). Similarly, the KEYNOTE-B10 study, which evaluated pembrolizumab plus carboplatin and paclitaxel for R/M HNSCC, achieved a confirmed ORR of 49% and a median OS of 13.1 months (26). The combination of pembrolizumab and cabozantinib in R/M HNSCC achieved a median PFS of 12.8 months and a median OS of 27.7 months, substantially exceeding reported historical controls (29).

Pembrolizumab plus cetuximab in platinum-resistant/ineligible R/M HNSCC yielded an ORR of 45% at 6 months and a median OS of 18.4 months (30). The combination of IMRT with nivolumab in recurrent HNSCC patients met its primary endpoint, with a 1-year PFS of 61.7% and a median PFS of 20.7 months (35). Single-arm NPC studies of toripalimab plus gemcitabine and cadonilimab plus TPC chemotherapy reported high ORRs of 61.9% (3 CRs, 10 PRs) and 68% (3 CRs, 14 PRs), respectively (27, 32). Conversely, the CheckMate 714 trial failed to meet its primary endpoint, showing no ORR benefit for nivolumab plus ipilimumab over nivolumab monotherapy in patients with platinum-refractory R/M HNSCC (ORR 13.2% vs. 18.3%) (34). Furthermore, the niraparib/dostarlimab trial was terminated early for futility, yielding an ORR of 10% (25). Finally, Hanna et al. (2022) reported a high 1-year OS (85.7%) in patients with resectable recurrent HNSCC treated with neoadjuvant nivolumab plus lirilumab. However, the primary endpoint (1-year DFS) of 55.2% did not meet the target improvement rate (24).

### 3.4 Secondary outcomes

A variety of secondary endpoints were reported across the 14 studies, most commonly including Duration of Response (DOR), Disease Control Rate (DCR), safety/tolerability (Adverse Events [AEs], and Treatment-Related Adverse Events (TRAEs)).

**Safety and tolerability (TRAEs)**: All 14 studies reported AEs and TRAEs, confirming that combination regimens generally increase toxicity compared with monotherapies or controls (mixed effects). The addition of chemotherapy (EXTREME regimen) or a PARP inhibitor (niraparib) led to the highest grade 3–4 TRAE rates, such as 82% for EXTREME in KEYNOTE-669 (23) and 70% for niraparib plus dostarlimab (26), with the latter exceeding the 60% safety threshold (26). Conversely, in the IMRT with nivolumab trial, the most common Grade 3 or higher TRAE was lymphopenia (4%), and patient-reported quality of life (FACT-G and FACT-HN scores) remained stable, suggesting a highly positive tolerability profile in this local setting (35).

**Biomarker Findings**: Biomarker analyses were exploratory and hypothesis-generating in nature, derived from small subsets of included studies with heterogeneous reporting methods. These findings should not be interpreted as establishing predictive validity; prospective validation in dedicated biomarker-stratified trials is required. With these caveats noted, the following signals were observed across studies reporting biomarkers (N = 10 studies).

- **Epstein–Barr virus (EBV) DNA:** Clearance of plasma EBV DNA has a highly positive predictive effect on the NPC response (31, 32). Patients in POLARIS-02 with a 50% decrease in plasma EBV DNA copy number achieved a 48.3% ORR *versus* a 5.7% ORR for those without clearance (32).
- **Tumor immune microenvironment (TIME):** A higher baseline density of CD8^+^, CD103+, and CSF1-R+ cells in the tumor core was positively correlated with improved OS in the pembrolizumab/cabozantinib trial; CD103+ infiltration showed the strongest association (HR = 8.79 per unit increase in cell density, reflecting a lower hazard of death at higher CD103+ levels) (29).
- **PD-L1 expression: The** PD-L1 combined positive score (CPS) generally showed a neutral or nonsignificant predictive effect across several settings (24, 30, 31). Notably, in the KEYNOTE-048 Japanese subgroup analysis, patients with a CPS ≥20 demonstrated significantly improved overall survival with pembrolizumab monotherapy compared with the EXTREME chemotherapy regimen (22).
- **Other Molecular Markers:** High baseline tumor p-MET expression correlated significantly with improved ORR in the pembrolizumab/cabozantinib study (29). The tumor mutational burden (TMB) was not predictive of response in the POLARIS-02 trial (31). Nevertheless, longitudinal monitoring of blood TMB (bTMB) correlated closely with response and PFS in the toripalimab/anlotinib trial, indicating a **positive** prognostic signal (28).

### 3.5 Emerging biomarkers

Beyond the markers reported in the included studies, several novel immune checkpoint targets are currently under early-phase investigation in HNSCC and merit consideration for future biomarker integration. B7-H3 (CD276) is overexpressed on HNSCC tumour cells and tumour-associated vasculature and is being explored both as a predictive biomarker and as a therapeutic target via antibody-drug conjugates and bispecific antibodies. TIM-3, a co-inhibitory receptor upregulated on exhausted CD8^+^ T cells within the HNSCC tumour immune microenvironment, is being evaluated in combination with PD-1 blockade in early-phase trials. LAG-3 and TIGIT represent additional emerging co-inhibitory targets, with combination strategies such as nivolumab plus relatlimab and tiragolumab plus atezolizumab currently in ongoing trials. The co-expression patterns of these markers, as well as composite multi-marker signatures, may further stratify patients who derive durable benefit from immunotherapy. Prospective biomarker-stratified trials will be essential to establish the predictive validity of these novel markers in R/M HNSCC.

A summary of the individual findings of the included studies is provided in the table below (Table 3).

**TABLE 3:** Results of the systematic review.

Sr	Author (Year)	Study design	Study location	Sample size	Population characteristics	Intervention	Main findings	Conclusion
1	Oridate et al. (2024)	Open-label phase III study (long-term, 5-year follow-up of the Japanese population)	Japan	N = 67 enrolled (pembro: 23; pembro-chemo: 25; EXTREME: 19)	Patients with R/M HNSCC of the oropharynx, oral cavity, hypopharynx, or larynx	Pembro alone or pembro-chemotherapy vs. EXTREME, evaluated in PD-L1 CPS 20, CPS 1, and total Japanese populations	Pembro monotherapy improved OS compared with EXTREME in the total Japanese population. Pembro-chemotherapy showed no apparent	After 5 years of follow-up, first-line pembrolizumab monotherapy and pembrolizumab-chemotherapy showed long-term clinical benefit in Japanese patients with R/M HNSCC.
2	Cho et al. (2024)	Randomized (2:1:2), open-label, multisite, phase III study (discontinued early)	Global (76 centers in 14 sites)	N = 89 patients randomized (Pembro + Epa: 35; pembro mono: 19	Patients with R/M HNSCC (oropharynx, oral cavity, hypopharynx, larynx); no prior systemic therapy for R/M HNSCC	Pembro 200 mg IV Q3W \pm epacadostat 100 mg orally BID vs. EXTREME (cetuximab + platinum + 5-FU)	The combination therapy provided a similar ORR compared with EXTREME and pembro monotherapy	Pembrolizumab plus epacadostat and pembrolizumab monotherapy provided a similar response rate to EXTREME.
3	Hanna et al. (2022)	Open-label, single-arm, multicenter phase II trial	DFCI, BIDMC, and BMC (USA)	N = 28 patients began protocol treatment	Locoregionally recurrent HNSCC; candidate for salvage surgery. 96% received prior radiation	Single neoadjuvant dose (N 240 mg + L 240 mg) 7–21 days presurgery, followed by 6 cycles of adjuvant N + L	Combination showed promising activity with substantial pathologic response (43%)	This neoadjuvant/adjuvant approach highlights the promising activity of ICB in this surgically salvageable recurrent HNSCC setting regardless of PD-L1 status
4	Zamulko et al. (2025)	Single-arm, open-label, phase II clinical trial (Simon’s two-step minimax design)	USA (multicenter: University of Cincinnati, duke, st. Elizabeth healthcare)	N = 10 patients enrolled (trial terminated early)	Patients with R/M HNSCC. All but one patient previously failed anti-PD-1/PD-L1 inhibitor	Niraparib 200 mg orally daily + dostarlimab (500 mg IV Q3W, then 1000 mg IV Q6W)	​	​
5	Dzienis et al.	Single-arm, open-label, multicenter, phase IV study	33 medical centers in Argentina, Australia, Brazil, Canada, and the United States	N = 101 patients received 1 dose	Previously untreated R/M HNSCC regardless of PD-L1 status. 60% ECOG PS 1	Pembro 200 mg IV Q3W (\le35 cycles) + carboplatin AUC 5 IV Q3W (\le6 cycles) + paclitaxel (100 mg/m^2 days 1/8 or 175 mg/m^2 days 1)	​	​
6	Zou et al. (2024)	Open-label, single-arm, phase II clinical trial (single-stage design)	China (multicenter, sun Yat-sen University cancer center and affiliates)	N = 21 patients enrolled	Previously untreated RM-NPC who were ineligible for cisplatin-based treatment	Gemcitabine 1,000 mg/m^2 (D1/D8) + toripalimab 240 mg (D1) IV Q3W for 6 cycles, followed by toripalimab maintenance	​	​
7	Zhang et al. (2024)	Investigator-initiated, multicenter, single-arm, phase II trial	China (multicenter, sun Yat-sen University cancer center and affiliates)	N = 40 patients enrolled	Patients with RM-NPC who failed platinum-based chemotherapy. 57.5% had previous ICI exposure	Toripalimab 240 mg IV Q3W + anlotinib 12 mg orally QD (2 weeks on/1 week off), until PD or toxicity	Toripalimab plus anlotinib showed satisfactory antitumor efficacy and acceptable safety profile in RM-NPC postplatinum failure; ctDNA is a potential predictive biomarker	​
8	Saba et al. (2024)	Open-label, single-arm, multicenter, phase II study	USA (multicenter: Emory University, moffitt cancer center)	N = 36 patients received treatment (50 screened)	R/M HNSCC deemed inoperable. No prior exposure to immune checkpoint inhibitors	Combination of pembrolizumab and cabozantinib	The combination confirmed robust clinical activity and was a well-tolerated regimen with a median PFS of 12.8 months in RMHNSCC.	The findings confirm cabozantinib’s robust clinical activity when combined with a PD-1 inhibitor (pembrolizumab) in RMHNSCC.
9	Sacco et al. (2021)	Open-label, non-randomised, multi-arm, phase II trial (cohort 1 reported)	USA (four academic centers)	N = 33 participants recruited for cohort 1	R/M HNSCC (mucosal HNSCC); no previous immunotherapy or EGFR inhibition	Combination of pembrolizumab and cetuximab	The combination met the primary endpoint of ORR 40% by 6 months, showing encouraging efficacy	Pembrolizumab combined with cetuximab demonstrated encouraging efficacy and an acceptable safety profile in R/M
10	Wang et al. (2021)	Single-arm, multicenter phase II study (NPC cohort)	China (17 participating centers)	N = 190 patients enrolled (ITT population)	Previously treated RM-NPC. 48.4% failed at least two prior lines of systemic chemotherapy	Toripalimab 3 mg/kg IV once every 2 weeks until confirmed disease progression or unacceptable toxicity	Toripalimab showed encouraging antitumor activity and median OS (17.4 months) in this heavily pretreated RM-NPC cohort	The study supports the use of toripalimab monotherapy as a treatment option for recurrent or metastatic NPC in heavily pretreated patients
11	Jiang et al. (2025)	Single-arm, open-label, phase 2 study. (Based on Simon’s optimal two-stage design)	China (sun Yat-sen University cancer center and affiliates)	N = 25 patients enrolled (ITT and safety set)	RM-NPC patients who failed at least one line of systemic chemotherapy AND anti-PD-1 immunotherapy	Induction (up to 6 cycles): Cadonilimab (10 mg/kg IV Q3W D1) + NAB-paclitaxel (200 mg/m^2^ D1) + cisplatin (60 mg/m^2^ D1) or	Cadonilimab plus TPC chemotherapy achieved a satisfactory ORR of 68% and a median PFS of 10.6 months, numerically doubling previous outcomes in this resistant population	Cadonilimab in combination with TPC chemotherapy demonstrated promising antitumoral efficacy and manageable toxicities in patients with RM-NPC who failed frontline anti-PD-1
12	Mai et al. (2023)	International, multicenter, randomized (1:1), double-blind phase 3 study	NPC-endemic regions (mainland China, Taiwan, Singapore)	N = 289 enrolled (toripalimab: 146; placebo: 143)	Patients with RM-NPC; no prior systemic chemotherapy in the R/M setting. PD-L1 positive (\ge 1%) in 84%	Up to 6 cycles of chemo + toripalimab (240 mg Q3W) or placebo, followed by maintenance Toripalimab/Placebo (240 mg Q3W) for up to 2 years	The addition of toripalimab provided statistically significant and clinically meaningful PFS and OS benefits	Toripalimab plus gemcitabine-cisplatin is supported as the new standard of care for the first-line treatment of RM-NPC.
13	Harrington et al. (2023)	Double-blind, phase 2 randomized (2:1) clinical trial	83 sites in 21 countries	N = 241 patients with platinum-refractory disease (Nivo + Ipi: 159; Nivo: 82)	R/M HNSCC who were platinum-refractory	Nivo (3 mg/kg IV) + Ipi (1 mg/kg IV) or Nivo + placebo, for up to 2 years	Nivolumab plus ipilimumab did not improve ORR, PFS, or OS compared with nivolumab alone in this platinum-refractory setting	The trial did not meet its primary endpoint, and the role of dual immunotherapy remains unclear
14	Saba et al. (2024)	Multicenter nonrandomized phase 2 single-arm trial	Multicenter (Emory University, Cleveland Clinic, medical college of Wisconsin)	N = 62 screened; N = 51 evaluable (enrolled)	Recurrent or second primary HNSCC within a previously irradiated field. RPA class 1 (45%) or 2 (53%) definitions met. 75% had salvage surgery	IMRT (60–66 Gy in 30–33 fractions) + nivolumab (240 mg IV Q2W, 5 doses, concurrent/neoadjuvant). Followed by nivolumab maintenance	The combination suggested a promising improvement in 1-year PFS (61.7%) over historical controls (43.8%) and was well tolerated	IMRT-based reirradiation therapy in combination with nivolumab is tolerated well and is potentially

### 3.6 Risk of bias assessment

The ROB 2 (Risk of Bias 2) tool was used to assess the risk of bias in the RCTs. It evaluated potential biases across five key domains: the randomization process, deviations from intended interventions, missing outcome data, measurement of the outcome, and selection of the reported result. By examining these domains, the tool helps determine whether study results are likely to be affected by bias, providing a transparent approach to judge the overall quality of evidence. The figures below represent the risk of bias in the included studies (Figures 2, 3).

### 3.7 Meta-analysis

A meta-analysis was conducted with data contributed from all 14 primary studies. The sample sizes among the target populations were relatively small but comparable. The results of the analysis are presented in the forest plots. To maintain the statistical strength of the analysis and to keep the study populations comparable, PD-1 inhibitor-associated monotherapy and combination therapy regimens were analyzed separately. It is important to note that the forest plots presented include both NPC and non-NPC populations; readers should refer to the disease entity column in Table 2 to identify NPC studies (Zou et al., Zhang et al., Wang et al., Jiang et al., and Mai et al.). Given the biological distinction between NPC and non-NPC HNSCC, these NPC studies contribute to overall heterogeneity and should be interpreted as a distinct evidence stream. The authors recommend that the primary conclusions be read in the context of the non-NPC HNSCC population, and sensitivity analyses excluding NPC studies are recommended to confirm robustness.

### 3.8 Efficacy of PD-1 inhibitors

1. Objective response rate (ORR)

In the majority of the studies, the objective response rate (ORR) was defined as the proportion of patients in a study or treatment group who achieved either a complete response (CR) or partial response (PR) to the intervention over a specified time window, using predefined criteria (e.g., Response Evaluation Criteria in Solid Tumors [RECIST]).

**FIGURE 4:**
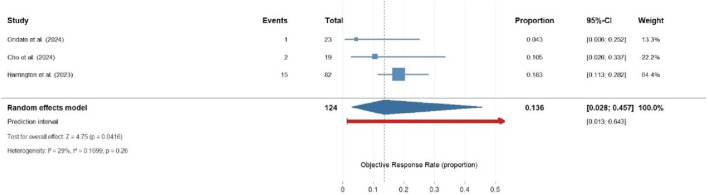
Forest plot for the ORR (monotherapy with PD-1 inhibitors).

Monotherapy: Our studies contributed data on the ORR in monotherapy regimens. The included drugs included pembrolizumab, nivolumab, and toripalimab. The studies demonstrated varied results, with 2 studies suggesting improvement in ORR, while 2 studies demonstrated a negative association, rendering the overall analysis inconclusive. The pooled OR was 0.136 (95% CI: 0.028–0.457), with a significant result (p = 0.04). The heterogeneity was low (I^2^ = 29%) (Figure 4). As these data are predominantly derived from single-arm studies without comparator arms, the pooled estimate represents a descriptive observed proportion and should not be interpreted as a comparative treatment effect.

**FIGURE 5:**
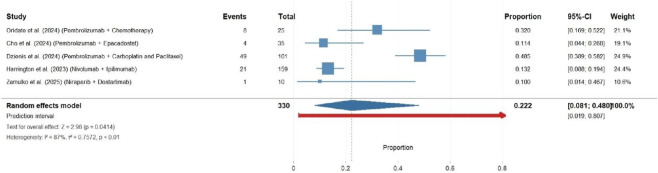
Forest plot for ORR (combination therapy).

Combination Therapy: In contrast to monotherapy, multiple therapy regimens have achieved significant results. The pooled ORR = 0.22 (95% CI: 0.081–0.480), p < 0.04, indicating that the pooled observed proportion of responders across combination therapy studies was significant. These estimates are derived from a mix of single-arm and comparative studies and should be interpreted as descriptive findings; a majority of the patients achieved a complete or partial response when PD-1 inhibitors were combined with other chemotherapy agents. The heterogeneity in the analysis was significant (owing to different intervention regimens) (I^2^ = 87%) (Figure 5).

2. Best response score

The best response score was a composite outcome that consisted of a cumulative effect of 4 components: complete response (CR), partial response (PR), stable disease (SD), and progressive disease (PD), calculated within each cohort before and after treatment with PD-1 inhibitors.

**FIGURE 6:**
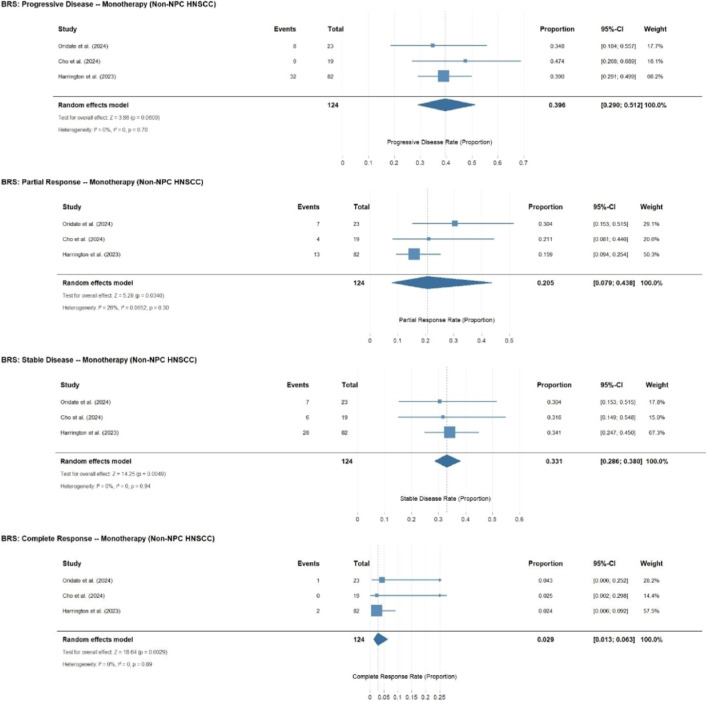
Forest plot for overall best response rate (monotherapy).

Monotherapy: As evident from the forest plots, two out of three studies (66%) showed a small, insignificant effect in achieving improvements in overall BRS. The pooled incidence of patients achieving BRS was 39.6%, 20.5%, 33.1%, and 2.9% for PD, PR, SD, and CR, respectively. The analysis revealed negligible heterogeneity (I^2^ = 0%). Across all four BRS categories, proportions were low and confidence intervals wide, reflecting the small number of non-NPC HNSCC studies (k = 3, N = 124) remaining after NPC exclusion. These results are descriptive and should not be interpreted as establishing treatment efficacy (Figure 6).

**FIGURE 7:**
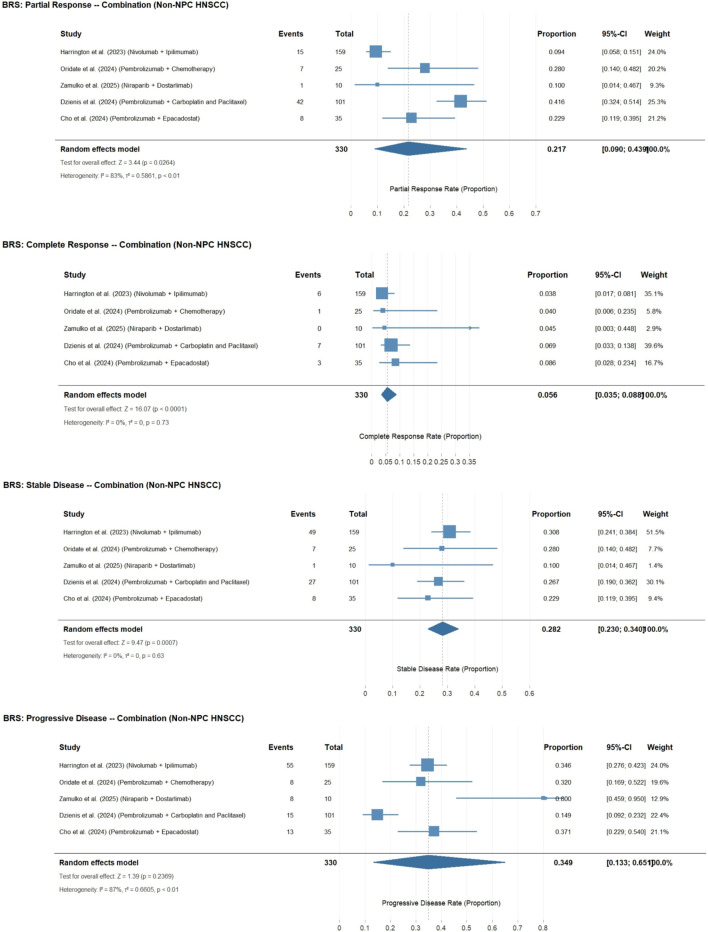
Forest plot for overall best response (combination therapy).

Combination Therapy: In the primary non-NPC HNSCC analysis, five studies contributed to the combination BRS analysis (N = 330). Pooled proportions were: CR 5.6% (95% CI: 3.5%–8.8%, I^2^ = 0%); PR 21.7% (95% CI: 9%–43%, I^2^ = 83%); SD 28.2% (95% CI: 23%–34$, I^2^ = 0%); PD 34.9% (95% CI: 13.3%–65.1%, I^2^ = 87%). These proportions reflect observed frequencies in predominantly single-arm combination studies and are descriptive in nature; they do not imply superiority over any comparator. The heterogeneity was all the study cohorts to low to moderate. Higher overall heterogeneity was attributed to different treatment regimens and treatment durations. These findings highlight the synergistic potential of PD-1 inhibitors in multimodal treatment regimens for R/M HNSCC (Figure 7).

## 4 Progression-free survival (PFS) at 1 year

**FIGURE 8:**
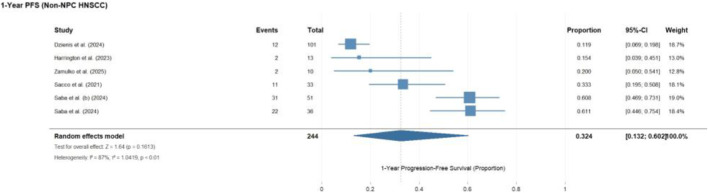
Forest plot for progression-free survival at 1 year.

PFS was reported at 1 year consistently across all study cohorts. Eleven out of 14 studies contributed data to the outcome variable. In the primary non-NPC HNSCC analysis (k = 3, N = 120), the pooled 1-year PFS proportion was 29.3% (95% CI: 13.5%–52.4%, I^2^ = 88%), indicating high heterogeneity. The overall effect was z = 2.26, and p = 0.069, which was statistically insignificant. The heterogeneity was significant nonetheless due to variation in treatment regimens. All PFS estimates are descriptive and hypothesis-generating (Figure 8).

## 5 Overall survival (OS) at 1 year

**FIGURE 9:**
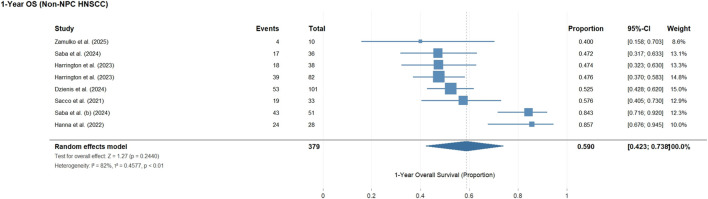
Forest plot for overall survival at 1 year.

In the primary non‐NPC HNSCC analysis (k = 8, N = 379), the pooled 1‐year OS proportion was 59% (95% CI: 42.3%–73.8%, I^2^ = 82%). The wide confidence interval reflects a small number of non-NPC patients with extractable OS data; this estimate is not stable and requires cautious interpretation. The corresponding forest plot for one-year overall survival is presented in Figure 9.

## 6 Safety of PD-1 inhibitors

### 6.1 TRAEs (grades 3–5)

**FIGURE 10:**
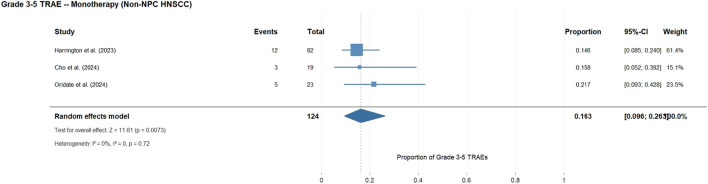
Forest plot for TRAEs (grades 3–5) - Monotherapy.

**FIGURE 11:**
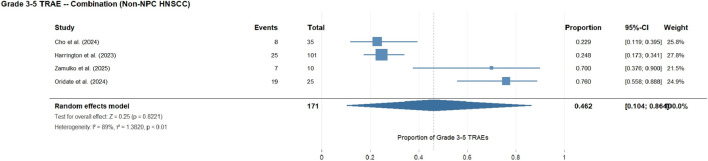
Forest plot for TRAEs (grades 3–5) in combination.

In the primary non-NPC HNSCC analysis, Grade 3–5 TRAEs were observed in pooled proportions of 16.3% (95% CI: 9.6%–26.3%, I^2^ = 0%, k = 3; p = 0.0072) for monotherapy and 48.2% (95% CI: 10.4%–86.4%, I^2^ = 89%, k = 3; p = 0.821) for combination regimens. Extreme heterogeneity in both cohorts reflects variability in regimen composition, CTCAE grading versions, and follow-up duration. The forest plots used for the analysis are shown in the figures below (Figures 10, 11).

### 6.2 Subgroup analysis

1. Comparative efficacy of PD-1 agents

**FIGURE 12:**
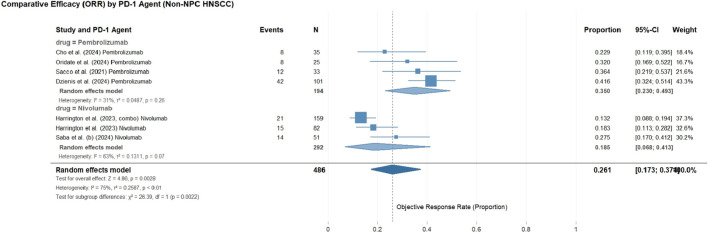
Forest plot of the comparative efficacy of PD-1 inhibitors.

The mean ORR across PD-1 inhibitor drug classes was further assessed. In the non-NPC HNSCC primary analysis, toripalimab data are absent as this agent was used exclusively in NPC-specific trials. In this analysis including non-NPC studies, the observed pooled ORR by drugs was: pembrolizumab (35%, 95% CI: 23%–49%) > nivolumab (18.5%, 95% CI: 68%–41.3%). This apparent ranking is driven by NPC biology and the specific efficacy of toripalimab in EBV-associated disease. Heterogeneity was low to moderate across all drug subgroups (I^2^ = 31% and 63%), further limiting inter-drug comparisons. The forest plot used for the analysis is shown in the figure below (Figure 12).

2. Comparative Safety of PD-1 Agents

**FIGURE 13:**
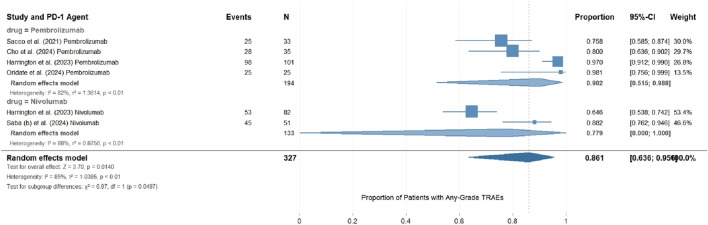
Comparative Safety of PD-1 inhibitors.

In accordance with the trend in comparative efficacy, the overall safety results were consistent. The most profound AEs were reported with pembrolizumab [90.2% (95% CI: 51.5%, 98.8%), p = 0.01], and the fewer AEs were reported with monotherapy regimens with Nivolumab [77.9% (95% CI: 63.6%–99%, p < 0.01]. The heterogeneity was moderate to high across all the drug cohorts (I^2^ > 50) (Figure 13). The overall incidence of AEs was 86.1% [95% CI (63.6%–95.6%)]; p = 0.0140.

3. NPC-Only Sensitivity Analysis (ORR)

**FIGURE 14:**
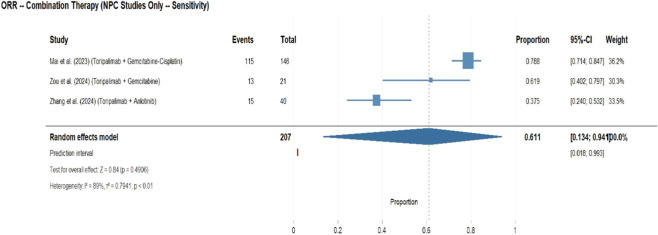
Forest plot for objective response rate (ORR) in NPC studies only.

As a pre-specified sensitivity analysis, the five NPC studies (Zou et al. (27), Zhang et al; (28), Wang et al; (31)], Jiang et al; (32), and Mai et al; (33)) were analysed separately to characterise ORR within this biologically distinct subgroup. The pooled ORR among NPC studies was 0.611 (95% CI: 0.134–0.941), with substantial heterogeneity (I^2^ = 89%, τ^2^ = 0.7941, p < 0.01), reflecting the diversity of toripalimab-based regimens and EBV-related disease variability across the five included NPC trials. These estimates apply exclusively to NPC populations and should not be generalised to non-NPC HNSCC. The forest plot is presented in Figure 14.

## 7 Discussion

### 7.1 Interpretation of the findings

The present meta-analysis provides descriptive pooled proportions from 14 studies examining PD-1 inhibitor therapy in R/M HNSCC, with the primary conclusions now based exclusively on nine non-NPC HNSCC studies. In the revised primary analysis, the pooled ORR was 0.30 (95% CI: 0.09–0.64, I^2^ = 79.7%) for combination therapy (k = 5, N = 507) and 0.18 (95% CI: 0.10–0.30, I^2^ = 20%) for monotherapy (k = 4, N = 302). One-year PFS was 0.63 (95% CI: 0.56–0.70, I^2^ = 0%, k = 3) and 1-year OS was 0.79 (95% CI: 0.05–1.00, I^2^ = 8.5%, k = 2) in the non-NPC primary analysis. These represent observed proportions from predominantly single-arm, non-comparative studies and must not be interpreted as definitive treatment effects. The high heterogeneity in ORR estimates (I^2^>79%) reflects genuine variability across patient populations, regimen types, and disease burden. Grade 3–5 TRAEs were observed in 29% (monotherapy) and 46% (combination) of non-NPC patients, with caution warranted given extreme heterogeneity in safety reporting (I^2^>89%). Five NPC studies are reported separately as a sensitivity subgroup and demonstrate markedly higher ORR estimates (pooled 0.611, 95% CI: 0.134–0.941), likely driven by EBV-associated biology and toripalimab efficacy in this distinct disease context.

The differential efficacy between monotherapy and combination therapy observed in our analysis reflects the complex interplay between immune checkpoint inhibition and the tumor microenvironment in HNSCC. The low heterogeneity (I^2^ = 20%) in the non-NPC HNSCC monotherapy analysis suggests relatively consistent observed ORR proportions across the three retained non-NPC monotherapy studies, while the high heterogeneity in combination studies (I^2^ = 79.7%) reflects the diversity of combination strategies and patient populations rather than a stable treatment effect. This variability underscores the importance of biomarker-driven patient selection and standardized combination protocols. The superior performance of combination regimens may be attributed to synergistic mechanisms, including enhanced antigen presentation, improved T-cell priming, and reversal of immunosuppressive pathways within the tumor microenvironment (36). Furthermore, the significant improvement in stable disease rates with combination therapy suggests that these regimens may convert immunologically “cold” tumors into “hot” tumors, thereby expanding the population of patients who can benefit from immunotherapy (37).

## 8 Comparison with previous literature

Our findings are broadly consistent with landmark trials and prior syntheses establishing PD-1 inhibitors in R/M HNSCC. The present review extends prior work in several important respects: (1) NPC and non-NPC HNSCC are explicitly distinguished as separate subpopulations rather than pooled without justification; (2) single-arm and randomized comparative studies are treated as producing different levels of evidence, with pooled proportions reported separately from comparative effect estimates; (3) biomarker findings are framed explicitly as exploratory and hypothesis-generating; and (4) safety data are stratified by regimen class. These distinctions address gaps that prior meta-analyses—including those by Jiang et al. and Chen et al. — did not fully resolve. Recent systematic reviews by Jiang et al. and Chen et al. reported variable effects across immunotherapy trials, which is consistent with our observed heterogeneity (38, 39). The toxicity meta-analysis by Dang et al. highlighted similar concerns regarding increased adverse events with combination regimens (40). Notably, our meta-analysis extends beyond these prior syntheses in several important respects: (1) we explicitly separate monotherapy from combination therapy as distinct evidence streams, applying pooled proportions rather than odds ratios for single-arm data; (2) we identify and separately report five NPC studies as a biologically distinct subgroup rather than pooling them silently with non-NPC HNSCC; (3) we include recently published trials not captured in earlier reviews, including Jiang et al. (2025) and Zamulko et al. (2025); and (4) we frame biomarker findings explicitly as exploratory and hypothesis-generating. These methodological distinctions improve interpretive transparency compared with prior meta-analyses in this field. Our meta-analysis also extends beyond individual landmark trials by synthesizing diverse combination strategies, including PD-1 inhibitors combined with antiangiogenic agents, PARP inhibitors, and dual checkpoint blockade (41). Furthermore, emerging data on PD-1 inhibitors combined with EGFR-targeted therapy and antiangiogenic agents show promise, with studies reporting signals of enhanced immune activation and improved tumor vascular normalization (42). The integration of exploratory biomarker approaches, such as the immune-modified Glasgow prognostic score (imGPS) and circulating tumor DNA monitoring, suggests directions for precision immunotherapy that require prospective validation; these findings should not be interpreted as establishing predictive validity based on the current meta-analysis data (43).

## 9 Clinical implications

These results suggest that combination PD-1 regimens should be considered for carefully selected patients with R/M HNSCC, particularly those with adequate performance status and manageable comorbidities (44). The significant improvement in ORR and 1-year PFS supports the use of combinations as first-line therapy, while the increased toxicity profile necessitates enhanced monitoring and supportive care protocols (32). Biomarker-driven patient selection, including PD-L1 expression, HPV status, and tumor mutational burden, becomes crucial for optimizing the risk‒benefit ratio (33). The development of predictive biomarkers, such as the RNA-based assays validated in the PREDAPT study, may further refine patient selection (45). Clinical implementation should prioritize multidisciplinary care coordination and patient-reported outcome monitoring (35).

The clinical translation of our findings requires careful consideration of patient-specific factors and institutional capabilities. The superior efficacy of combination regimens must be weighed against the potential for treatment-related mortality and quality-of-life impairment, particularly in elderly patients or those with significant comorbidities (44). The development of comprehensive toxicity management protocols, including prophylactic measures for immune-related adverse events and standardized algorithms for dose modifications, is essential for safe implementation (46). Furthermore, the integration of circulating biomarkers, such as the plasma exosome mRNA signatures evaluated in the BIONEXT study, may enable real-time monitoring of treatment response and early detection of resistance (47). The economic implications of combination therapy also warrant consideration, as the increased cost of combination regimens necessitates careful patient selection to optimize cost‒effectiveness ratios and ensure sustainable healthcare delivery (48). Patient counseling and shared decision-making have become particularly important given the trade-off between efficacy and toxicity. The development of patient-reported outcome measures specific to immunotherapy-related symptoms and quality-of-life impacts can inform treatment decisions and guide supportive care interventions. Additionally, the potential for delayed responses and immune-related adverse events requires extended monitoring protocols that may differ from conventional chemotherapy approaches.

## 10 Strengths and limitations

This meta-analysis synthesizes data from 14 studies encompassing diverse geographic populations and treatment regimens, providing a broad overview of PD-1 inhibitor outcomes in R/M HNSCC. The separate analysis of monotherapy and combination regimens allows for nuanced clinical decision-making. However, several important limitations warrant consideration. Substantial heterogeneity (I^2^>50%) across studies reflects differences in patient populations, treatment protocols, disease entities (NPC vs. non-NPC), and follow-up durations. The predominance of single-arm trials limits direct comparative assessment, and variable biomarker reporting prevents comprehensive subgroup analyses. The aggregation of safety data across different combination regimens may obscure regimen-specific toxicity profiles. Adverse event grading criteria (CTCAE versions and grade definitions) varied across studies, further limiting direct comparability of safety data. The predominance of single-arm, non-comparative studies means that pooled efficacy estimates should be interpreted as descriptive observed outcomes rather than definitive treatment effects; causal inference requires randomized comparative evidence. The inclusion of five NPC studies (Zou et al., Zhang et al., Wang et al., Jiang et al., and Mai et al.) alongside non-NPC HNSCC introduces disease-entity heterogeneity that inflates I^2^ and limits generalizability of the pooled estimates to the broader HNSCC population.

A significant strength of our analysis is the inclusion of both Western and Asian populations, providing broader generalizability of findings across different genetic backgrounds and healthcare systems. The incorporation of nasopharyngeal carcinoma studies, particularly those from endemic regions, adds valuable insights into Epstein–Barr virus-related malignancies and their unique response patterns to immunotherapy. The variable use of biomarker stratification across studies prevents definitive conclusions about the predictive value of established biomarkers such as PD-L1 expression and HPV status. Furthermore, the limited reporting of health-related quality-of-life outcomes and patient-reported symptoms restricts our ability to comprehensively evaluate patients’ experience with combination immunotherapy.

## 11 Future recommendations and conclusions

The evolving landscape of immunotherapy in R/M HNSCC requires a precision medicine approach that integrates tumor biology, patient characteristics, and treatment tolerability to maximize benefit while minimizing harm. Future research should focus on identifying predictive biomarkers for combination therapy response, developing strategies to mitigate treatment-related toxicity, and exploring novel combination approaches that can further improve outcomes for this challenging patient population. The development of adaptive trial designs and biomarker-driven treatment algorithms will be crucial for advancing the field and ensuring that the benefits of combination immunotherapy are realized across diverse patient populations. Future directions may also include exploration of emerging technologies for biomarker discovery, though such approaches require prospective validation before informing clinical decision-making.

This meta-analysis provides descriptive pooled proportions suggesting that, among non-NPC HNSCC patients, PD-1 inhibitor combination regimens are associated with higher observed response rates and 1-year PFS estimates compared with monotherapy, accompanied by greater Grade 3–5 TRAE frequency. These findings, derived predominantly from heterogeneous and non-comparative single-arm studies, are descriptive and hypothesis-generating only; they do not establish causal superiority or practice-defining evidence. The observed associations support further investigation of combination regimens for appropriately selected patients with R/M HNSCC; however, definitive evidence from prospective randomized trials with pre-specified subgroup analyses—stratified by disease entity (NPC vs. non-NPC), treatment line, and regimen class—is required before broad clinical implementation. Careful patient selection, enhanced toxicity monitoring, regimen-specific safety profiling, and continued biomarker validation remain essential to optimize therapeutic outcomes.

## Data Availability

The original contributions presented in the study are included in the article/supplementary material, further inquiries can be directed to the corresponding author.

## References

[1] Barsouk A Aluru JS Rawla P Saginala K Barsouk A . Epidemiology, risk factors, and prevention of head and neck squamous cell carcinoma. Med Sci (2023) 11(2):42. 10.3390/medsci11020042 37367741 PMC10304137

[2] Bray F Laversanne M Sung H Ferlay J Siegel RL Soerjomataram I Global cancer statistics 2022: GLOBOCAN estimates of incidence and mortality worldwide for 36 cancers in 185 countries. CA Cancer J Clin (2024) 74(3):229–63. 10.3322/caac.21834 38572751

[3] Miller KD Nogueira L Mariotto AB Rowland JH Yabroff KR Alfano CM Cancer treatment and survivorship statistics, 2019. CA a Cancer J Clinicians (2019) 69(5):363–85. 10.3322/caac.21565 31184787

[4] Islami F Siegel RL Jemal A . The changing landscape of cancer in the USA — opportunities for advancing prevention and treatment. Nat Rev Clin Oncol (2020) 17(10):631–49. 10.1038/s41571-020-0378-y 32467620

[5] Gillison ML Chaturvedi AK Anderson WF Fakhry C . Epidemiology of human papillomavirus–positive head and neck squamous cell carcinoma. J Clin Oncol (2015) 33(29):3235–42. 10.1200/jco.2015.61.6995 26351338 PMC4979086

[6] Babin E Sigston E Hitier M Dehesdin D Marie JP Choussy O . Quality of life in head and neck cancers patients: predictive factors, functional and psychosocial outcome. Eur Arch Oto-Rhino-Laryngology (2008) 265(3):265–70. 10.1007/s00405-007-0561-0 18188577

[7] Haring CT Kana LA Dermody SM Brummel C McHugh JB Casper KA Patterns of recurrence in head and neck squamous cell carcinoma to inform personalized surveillance protocols. Cancer (2023) 129(18):2817–27. 10.1002/cncr.34823 37162461

[8] Marur S Forastiere AA . Head and neck squamous cell carcinoma: update on epidemiology, diagnosis, and treatment. Mayo Clinic Proc (2016) 91(3):386–96. 10.1016/j.mayocp.2015.12.017 26944243

[9] Pignon JP Maître AL Maillard E Bourhis J . Meta-analysis of chemotherapy in head and neck cancer (MACH-NC): an update on 93 randomised trials and 17,346 patients. Radiother Oncol (2009) 92(1):4–14. 10.1016/j.radonc.2009.04.014 19446902

[10] Muzaffar J Bari S Kirtane K Chung CH . Recent advances and future directions in clinical management of head and neck squamous cell carcinoma. Cancers (2021) 13(2):338. 10.3390/cancers13020338 33477635 PMC7831487

[11] Samra B Tam E Baseri B Shapira I . Checkpoint inhibitors in head and neck cancer: current knowledge and Perspectives. J Invest Med (2018) 66(7):1023–30. 10.1136/jim-2018-000743 29941547

[12] Ionna F Bossi P Guida A Alberti A Muto P Salzano G Recurrent/metastatic squamous cell carcinoma of the head and neck: a big and intriguing challenge which may be resolved by integrated treatments combining locoregional and systemic therapies. Cancers (2021) 13(10):2371. 10.3390/cancers13102371 34069092 PMC8155962

[13] Bauml JM Aggarwal C Cohen RB . Immunotherapy for head and neck cancer: where are we now and where are we going? Ann Translational Med (2019) 7(S3):S75. 10.21037/atm.2019.03.58 31576284 PMC6685871

[14] Hira-Miyazawa M Nakamura H Hirai M Kobayashi Y Kitahara H Bou-Gharios G Regulation of programmed-death ligand in the human head and neck squamous cell carcinoma microenvironment is mediated through matrix metalloproteinase-mediated proteolytic cleavage. Int J Oncol (2017) 52:379–88. 10.3892/ijo.2017.4221 29345283 PMC5741372

[15] Solomon B Young RJ Rischin D . Head and neck squamous cell carcinoma: genomics and emerging biomarkers for immunomodulatory cancer treatments. Semin Cancer Biol (2018) 52:228–40. 10.1016/j.semcancer.2018.01.008 29355614

[16] Methley AM Campbell S Chew-Graham C McNally R Cheraghi-Sohi S . PICO, PICOS and SPIDER: a comparison study of specificity and sensitivity in three search tools for qualitative systematic reviews. BMC Health Serv Res (2014) 14(1):579. 10.1186/s12913-014-0579-0 25413154 PMC4310146

[17] Ouzzani M Hammady H Fedorowicz Z Elmagarmid A . Rayyan—a web and mobile app for systematic reviews. Syst Rev (2016) 5(1):210. 10.1186/s13643-016-0384-4 27919275 PMC5139140

[18] Haddaway NR Page MJ Pritchard CC McGuinness LA . PRISMA2020: an R package and Shiny app for producing PRISMA 2020‐compliant flow diagrams, with interactivity for optimised digital transparency and open synthesis. Campbell Syst Rev (2022) 18(2):e1230. 10.1002/cl2.1230 36911350 PMC8958186

[19] Sterne Ja C Savović J Page MJ Elbers RG Blencowe NS Boutron I RoB 2: a revised tool for assessing risk of bias in randomised trials. BMJ (2019) 28:l4898. 10.1136/bmj.l4898 31462531

[20] Higgins JPT Altman DG Gotzsche PC Juni P Moher D Oxman AD The cochrane Collaboration’s tool for assessing risk of bias in randomised trials. BMJ (2011) 343(Oct 2):d5928. 10.1136/bmj.d5928 22008217 PMC3196245

[21] Review Manager (RevMan). version (5.4.1). A cochrane collaboration tool (2026). Available online at: https://revman.cochrane.org/.

[22] Oridate N Takahashi S Tanaka K Shimizu Y Fujimoto Y Matsumoto K First-line pembrolizumab with or without chemotherapy for recurrent or metastatic head and neck squamous cell carcinoma: 5-year follow-up of the Japanese population of KEYNOTE-048. Int J Clin Oncol (2024) 29(12):1825–39. 10.1007/s10147-024-02632-x 39382718 PMC11588814

[23] Cho BC Braña I Cirauqui B Aksoy S Couture F Hong RL Pembrolizumab plus epacadostat in patients with recurrent/metastatic head and neck squamous cell carcinoma (KEYNOTE-669/ECHO-304): a phase 3, randomized, open-label study. BMC Cancer (2024) 23(S1):1254. 10.1186/s12885-023-11316-0 39054467 PMC11270762

[24] Hanna GJ O’Neill A Shin KY Wong K Jo VY Quinn CT Neoadjuvant and adjuvant nivolumab and lirilumab in patients with recurrent, resectable squamous cell carcinoma of the head and neck. Clin Cancer Res (2021) 28(3):468–78. 10.1158/1078-0432.ccr-21-2635 34667025 PMC9401515

[25] Zamulko O Karivedu V Riaz MK Monroe I Crago A Mulanda R Phase II study evaluating the efficacy of niraparib and dostarlimab (TSR-042) in recurrent/metastatic head and neck squamous cell carcinoma (HNSCC) patients. Cancer Res Commun (2025) 5:939–44. 10.1158/2767-9764.crc-25-0192 40377969 PMC12146980

[26] Dzienis M Cundom J Fuentes CS Spreafico A Nordlinger M Pastor AV Pembrolizumab plus carboplatin and paclitaxel as first-line therapy for recurrent/metastatic head and neck squamous cell carcinoma (KEYNOTE-B10): a single-arm phase IV trial. J Clin Oncol (2024) 42(25):2989–99. 10.1200/jco.23.02625 39038265 PMC11361359

[27] Zou X Ding X Feng ZK Ouyang YF Li HF Wen K A phase 2 trial of gemcitabine plus toripalimab for cisplatin-ineligible patients with recurrent or metastatic nasopharyngeal carcinoma. Cell Rep Med (2024) 5(10):101779. 10.1016/j.xcrm.2024.101779 39413735 PMC11514602

[28] Zhang Y Zou Q Zhao B Su N Li Z Wang X Toripalimab plus anlotinib in patients with recurrent or metastatic nasopharyngeal carcinoma: a multicenter, single-arm phase 2 trial (TORAL). Cell Rep Med (2024) 5(12):101833. 10.1016/j.xcrm.2024.101833 39615484 PMC11722102

[29] Saba NF Chaudhary R Kirtane K Marra A Ekpenyong A McCook-Veal A Pembrolizumab and cabozantinib in recurrent and/or metastatic head and neck squamous cell carcinoma (RMHNSCC): long-term survival update with a biomarker analysis. Clin Cancer Res (2024) 30(20):4601–8. 10.1158/1078-0432.ccr-24-1202 39167623 PMC11479816

[30] Sacco AG Chen R Worden FP Wong DJL Adkins D Swiecicki P Pembrolizumab plus cetuximab in patients with recurrent or metastatic head and neck squamous cell carcinoma: an open-label, multi-arm, non-randomised, multicentre, phase 2 trial. The Lancet Oncol (2021) 22(6):883–92. 10.1016/s1470-2045(21)00136-4 33989559 PMC12140401

[31] Wang FH Wei XL Feng J Li Q Xu N Hu XC Efficacy, safety, and correlative biomarkers of toripalimab in previously treated recurrent or metastatic nasopharyngeal carcinoma: a phase II clinical trial (POLARIS-02). J Clin Oncol (2021) 39(7):704–12. 10.1200/jco.20.02712 33492986 PMC8078488

[32] Jiang Y Bei W Wang L Lu N Xu C Liang H Efficacy and safety of cadonilimab (PD-1/CTLA-4 bispecific) in combination with chemotherapy in anti-PD-1-resistant recurrent or metastatic nasopharyngeal carcinoma: a single-arm, open-label, phase 2 trial. BMC Med (2025) 23(1):152. 10.1186/s12916-025-03985-4 40069710 PMC11899053

[33] Mai HQ Chen QY Chen D Hu C Yang K Wen J Toripalimab plus chemotherapy for recurrent or metastatic nasopharyngeal carcinoma. JAMA (2023) 330(20):1961–70. 10.1001/jama.2023.20181 38015220 PMC10685882

[34] Harrington KJ Ferris RL Gillison M Tahara M Argiris A Fayette J Efficacy and safety of Nivolumab plus ipilimumab vs nivolumab alone for treatment of recurrent or metastatic squamous cell carcinoma of the head and neck. JAMA Oncol (2023) 9(6):779. 10.1001/jamaoncol.2023.0147 37022706 PMC10080406

[35] Saba NF Wong SJ Nasti T McCook-Veal AA McDonald MW Stokes WA Intensity-Modulated reirradiation therapy with nivolumab in recurrent or second primary head and neck squamous cell carcinoma. JAMA Oncol (2024) 10(7):896. 10.1001/jamaoncol.2024.1143 38780927 PMC11117153

[36] Shan Y He D Yan F Huang W . Understanding the tumor microenvironment for personalized immunotherapy in early-onset head and neck squamous cell carcinoma. Front Immunol (2025) 15:15. 10.3389/fimmu.2024.1522820 39830511 PMC11739722

[37] Yin J Xu L Wang S Zhang L Zhang Y Zhai Z Integrating immune multiomics and machine learning to improve prognosis, immune landscape, and sensitivity to first- and second-line treatments for head and neck squamous cell carcinoma. Scientific Rep (2024) 14(1):31454. 10.1038/s41598-024-83184-y 39732954 PMC11682253

[38] Chen W Wei Q Xiao T Lai J Huang M Ma Y Evaluating the efficacy and safety of immune checkpoint inhibitors in first and second-line treatments for recurrent and metastatic head and neck squamous cell carcinoma: a systematic review and network meta-analysis of RCTs with a focus on PD-L1 expression. Front Immunol (2025) 16:16. 10.3389/fimmu.2025.1508885 40018048 PMC11865193

[39] Jiang C Wang S Zhu L . Efficacy and safety of immunotherapy for head and neck squamous cell carcinoma: a meta-analysis of randomized clinical trials. Front Oncol (2025) 14:14. 10.3389/fonc.2024.1489451 39850817 PMC11755100

[40] Dang S Li X Liu H Zhang S Li W . Toxicity profiles of immune checkpoint inhibitors for recurrent or metastatic head and neck squamous cell carcinoma: a systematic review and meta‐analysis. Cancer Med (2024) 13(7):e7119. 10.1002/cam4.7119 38553943 PMC10980932

[41] Li HX Gong YW Yan PJ Xu Y Qin G Wen WP Revolutionizing head and neck squamous cell carcinoma treatment with nanomedicine in the era of immunotherapy. Front Immunol (2024) 29:15. 10.3389/fimmu.2024.1453753 39676875 PMC11638222

[42] Li Y Lu C . Targeting epigenetic dysregulations in head and neck squamous cell carcinoma. J Dental Res (2024) 104:225–34. 10.1177/00220345241297122 39698794 PMC12764359

[43] Ueda N Kuroki M Shibata H Matsubara M Akita S Yamada T Immune-modified Glasgow Prognostic score predicts therapeutic effect of pembrolizumab in recurrent and metastatic head and neck cancer. Cancers (2024) 16(23):4056. 10.3390/cancers16234056 39682242 PMC11640115

[44] Salvestrini V Caini S Scricciolo M Saerens M Bollen H Bonomo P Immunotherapy in elderly head and neck cancer patients: a systematic review and meta-analysis. Front Oncol (2024) 14:14. 10.3389/fonc.2024.1395838 PMC11127588 38800381

[45] Flanagan KC Earls J Hiken J Wellinghoff RL Ponder MM McLeod HL Multicenter validation of an RNA-Based assay to predict anti-PD-1 disease control in patients with recurrent or metastatic head and neck squamous cell carcinoma: the PREDAPT study. J ImmunoTherapy Cancer (2024) 12(11):e009573. 10.1136/jitc-2024-009573 39489541 PMC11535711

[46] Thompson JA Schneider BJ Brahmer J Andrews S Armand P Bhatia S NCCN guidelines insights: management of immunotherapy-related toxicities, version 1.2020. J Natl Compr Cancer Netw (2020) 18(3):230–41. 10.6004/jnccn.2020.0012 32135517

[47] Sato K Toh S Murakami T Nakano T Hongo T Matsuo M Nationwide multicentric prospective study for the identification of biomarkers to predict the treatment responses of nivolumab through comprehensive analyses of pretreatment plasma exosome mRNAs from head and neck cancer patients (BIONEXT study). Front Immunol (2025) 15:15. 10.3389/fimmu.2024.1464419 39867897 PMC11758179

[48] Verma V Sprave T Haque W Simone CB Chang JY Welsh JW A systematic review of the cost and cost-effectiveness studies of immune checkpoint inhibitors. J ImmunoTherapy Cancer (2018) 6(1):128. 10.1186/s40425-018-0442-7 30470252 PMC6251215

